# Onychomycosis in diabetic patients in Fako Division of Cameroon: prevalence, causative agents, associated factors and antifungal sensitivity patterns

**DOI:** 10.1186/s13104-016-2302-1

**Published:** 2016-11-22

**Authors:** Marvlyn Eba, Anna Longdoh Njunda, Rene Njikam Mouliom, Emmanuel Tebit Kwenti, Anold Nsoh Fuh, Gordon Takop Nchanji, Julius Atashili

**Affiliations:** 1 Department of Medical Laboratory Sciences, Faculty of Health Sciences, University of Buea, P.O. Box 63, Buea, Cameroon; 2 Regional Hospital Limbe, Limbe, South West Region Cameroon; 3 Department of Microbiology and Parasitology, Faculty of Science, University of Buea, P.O. Box 63, Buea, Cameroon; 4 Department of Public Health and Hygiene, Faculty of Health Sciences, University of Buea, P.O. Box 63, Buea, Cameroon

**Keywords:** Onychomycosis, Diabetic, Prevalence, Risk factors, Antifungal susceptibility, Fako, Cameroon

## Abstract

**Background:**

Onychomycosis is an infection of the nail unit by a fungus. This is a very common infection amongst diabetics. Its occurrence among diabetics in Fako division is unknown. In this study we provide information on the characteristics of onychomycosis in diabetics in Fako division, Cameroon.

**Methods:**

A cross-sectional descriptive and analytical hospital-based study was conducted in two diabetic clinics in the Buea and Limbe regional hospitals. We recruited 152 consenting diabetics into the study. Demographic, behavioural, and clinical data of patients were obtained through the use of structured questionnaires. Toenail, finger nail, skin scrapings and nail clippings were collected from participants, KOH mounts were prepared and observed under the microscope and cultured on Sabouraud Dextrose Agar supplemented with chloramphenicol to isolate causative fungi. Identification of isolates was done to species level using the cello tape flag method and slide culture. The presence of a dermatophyte by either microscopy or culture or both methods was considered positive for onychomycosis. Antifungal susceptibility testing was carried out using selected antifungals by the Kirby–Bauer disk diffusion method on Sabouraud Dextrose Agar.

**Results:**

Clinical onychomycosis was found in 77 of the 152 diabetics tested giving a prevalence of 50.7% (95% CI 42.4–58.9) in diabetics in Fako. No socio-demographic or clinical factor studied was significantly associated with onychomycosis. *Trichophyton rubrum* was the most common isolate (62%). Other isolates included *Trichophyton metagraphyte* (22%) and *Trichophyton tonsurans* (16%). Dermatophytes were sensitive to miconazole (66%), amphotericin B (19%) and ketoconazole (14%).

**Conclusion:**

Onychomycosis is common in diabetics in Fako signifying the need for regular screening by either microscopy or culture. Infected nails could be treated with miconazole.

**Electronic supplementary material:**

The online version of this article (doi:10.1186/s13104-016-2302-1) contains supplementary material, which is available to authorized users.

## Background

Onychomycosis refers to fungal infection of the nail unit which occurs in every part of the world. Studies have proven onychomycosis to be the most prevalent nail disease accounting for about 50% of all nail changes and constituting about 30% of all cutaneous fungal infections [1].

Diabetes mellitus (DM) is a clinical syndrome caused by a disorder in insulin secretion and/or action which results in metabolic changes, especially high blood glucose. DM is one of the major predisposing factors to onychomycosis. Due to the rising trend in the number of people becoming diabetic worldwide, WHO estimated that there will be 300 million diabetics by 2025 [2]. The overall risk ratio of diabetics having onychomycosis is 2.77 compared to age and sex matched non-diabetic controls [3] and the potential for serious sequelae is increased in diabetics with vascular disease of the extremity, peripheral neuropathy, and those using poorly fitting shoes. Nails with fungal infection may become thick and distorted, sometimes with sharp edges. Such nails can abrade or ulcerate adjacent skin leading to severe outcomes. The abrasion or ulceration can increase in size, become chronic, and act as a portal of infection for bacteria, fungi or other organisms. Impaired wound healing may result in increased morbidity, possible amputation of the lower extremity or even mortality. Diabetic patients with onychomycosis have a higher percentage of gangrene and/or foot ulcer (12.2%) compared to those without onychomycosis (3.8%), that is a threefold higher risk [4]. Thus nail infections represent a risk factor in diabetic patients because of possible sequels.

Most superficial fungal infections are caused by keratinophilic fungi (dermatophytes). The dermatophyte that is mostly implicated in onychomycosis and tinea pedis is *Trichophyton rubrum* [5]. Other species that mostly infect the nails are *Trichophyton mentagraphytes, Trichophyton tonsurans* and *Epidermophyton floccosum* with the latter being less isolated. Both dermatophytes and non-dermatophytes alongside *Candida albicans*, have been identified as sole etiologic agents of onychomycosis [6].

In onychomycosis, direct microscopy is the most efficient screening technique [3–5]. Culture is the only method by which the causative microorganism can be identified.

There are currently three oral agents prescribed for the treatment of onychomycosis: fluconazole, itraconazole, and terbinafine. These are considered the gold standards in terms of cure rates, as a meta-analysis showed >70% success rate with these agents.

In Cameroon, like in most countries in Sub-Saharan Africa, diabetes is also of public health importance with over 567,000 (prevalence of 6.5%) persons living with the disease. An estimated 15,000 deaths associated with diabetes was recorded in the country in 2015 [7].

Although national control programmes have been put forth to improve on the management of diabetes, occurrence of onychomycosis has not been investigated in these patients in the country probably due to inadequate knowledge on the disease, poverty and limited resources. Thus prevalence is unknown.

It is necessary to isolate the causative agents as variability is both geographic and, within a given region, temporal [4]. Organisms that cause clinically apparent disease tend to receive the most attention while pathogens whose invasion leads to hard-to-detect disease may be present in a region but are less likely to be identified.

Associated factors predisposing diabetics to onychomycosis vary based on the community in which they are found and also the activities common in that community. Though certain factors may cut across regions there are other factors that are region specific thus providing a need for investigating associated factors in Fako division.

There exist high rates of recurrence of onychomycosis as a result of relapse due to treatment failures or re-infection [5]. As such it is of principal significance for the antifungal susceptibility pattern of the isolated strains to be determined to reduce these high rates of recurrence and effective treatment of new cases.

Thus onychomycosis could be a significant cause of morbidity in Cameroon more especially to the vulnerable diabetics as well as other vulnerable groups such as patients with human immunodeficiency virus (HIV patients). The findings of this study will provide useful information needed for a better understanding of onychomycosis in diabetics in Fako division and Cameroon as a whole.

With the goal of providing information on the characteristics of onychomycosis in diabetics in Fako, we assessed the prevalence, associated factors, causative agents and the antifungal sensitivity patterns of the isolates.

## Methods

### Study design and settings

We conducted a cross-sectional descriptive and analytical hospital-based study from May to August 2014 at the Diabetic Clinics of the Regional hospital of Buea (RHB) and the Regional hospital of Limbe (RHL) found in Buea and Limbe respectively. Buea and Limbe constitute the two major cities of Fako division. These institutions serve as secondary referral hospitals for other hospitals and health institutions- public, faith based and private-in the region and beyond. They also serves as teaching hospitals to several university related health institutions within the region and beyond. These hospitals receive diabetic patients with both Types I and II diabetes of all ages every day of the week but specific days of the week are set as clinic days with specialist consultations and patient education being carried out.

### Participants and sampling

The study participants constituted attendants of the diabetic clinics of the RHB and RHL. To be eligible, participants had to be diabetic of age 21 years or more. All participants were examined by a dermatologist for signs of onychomycosis. Participants with gestational diabetes, two foot amputation and those who had been on antifungal treatment during the preceding four weeks were excluded. Convenient and consecutive sampling was used to select participants once they agreed to participate.

### Data collection, variables and measurement

A structured questionnaire (see Additional file 1: Questionnaire) was used to collect data from each participant. This questionnaire was used to assess demographic associated factors (age, gender, diabetic clinic attended), behavioural factors (occupation, level of education, hobbies, foot wear and nail cutting habits), and clinical factors (duration of diabetes, presence of foot ulcer, other co-morbid disease, amputation and structural deformity, family history of onychomycosis, trauma, clinical manifestation of onychomycosis). Data collection was carried out by the investigator (a trained microbiologist) and this minimised potential bias.

### Specimen collection

Nail specimens were collected from participants with suspected nail lesions. Severity of nail damage was classified as mild (<25% involvement or <4 nails involved), moderate (26–74% involvement or 5–8 nails involved) and severe (≥75% involvement or ≥9 nails involved). Where one or more of the toenails appeared clinically abnormal, then the two toenails that are clinically most likely to have onychomycosis were sampled. To obtain nail specimens from dystrophic nails, cleansing of the nail area with 90% alcohol was carried out to remove contaminants such as bacteria, any discoloured, dystrophic or brittle parts of the nail and affected nail was cut superficially as far back as possible using a No. 22 surgical blade including any crumbly material. In case of superficial involvement (as in white superficial onychomycosis) nail scrapings were collected and the free edge of the nail plate was also used for investigation. A separate sample was collected if there appeared to be fingernail involvement as well as dermatophyte infection on another part of the body of any patient. Nail specimens were scraped directly onto black carbon papers, which made it easier to see how much material had been collected, carefully folded and placed inside white envelopes and sealed providing ideal conditions for transportation to the laboratory. The envelopes containing samples were carefully labeled with patient code, age, sex, date, place and time of collection. Analysis was carried out within 24 h of specimen collection.

### Sample processing

Five to six small fragments (1–2 mm) or scrape material from nails was placed on a glass slide containing a drop of 10% Potassium Hydroxide and glycerol (10% KOH + glycerol), covered with a cover-slip, gently heated by passing through a flame 2 or 3 times (not allowing solution to boil) and put aside to digest for at least 30 min at room temperature, then examined with ×10 and ×40 objectives for the presence of septate fungal hyphae. Negative specimens were kept and re-examined the next day to avoid reporting false-negative results because of delayed clearance and as need arose some slides were kept till culture results became available. The glycerol prevented the preparation from drying off and made the slides still readable after several days.

Approximately 20 representative small nail fragments from each participant irrespective of their microscopy results were scattered on Sabouraud Dextrose Agar (SDA) plates supplemented with chloramphenicol. The plates were sealed with proprietary tape to prevent air-borne contamination in the laboratory and incubated at room temperature (27 °C) for 7–14 days while visually monitoring of plate was done on a daily basis for fast growing fungi. Pure isolates were obtained by sub-culturing on new SDA plates and colonies growing out of the inoculation area were regarded as contaminants.

Identification of fungus grown on culture plates was based on the colony morphology, reverse pigmentation on SDA and the observation of sporulating fungus with Lactophenol cotton blue stain. Identification to species level was done using the cellotape flag method and the slide culture procedure as described elsewhere [8, 9].

Susceptibility testing was carried out using the Kirby–Bauer disk diffusion method endorsed by the Clinical Laboratory Standards Institute (CLSI) [10] on dried SDA plates. Briefly, a tiny portion of the fungal colony was emulsified in 4 ml of sterile physiological saline, mixed and the turbidity of the suspension compared with 0.5 McFarland standard. A sterile swab was used to streak the suspension on SDA plates in three directions, rotating the plate approximately 60° to ensure even distribution. The isolates were tested against five antifungal agents including Amphotericin B (20 mcg, Biogram™), Ketoconazole (10 mcg Biogram™), Miconazole (50 mcg, Bioanalyse^®^), Griseofulvin (10 mcg, Bioanalyse^®^) and Itraconazole (50 mcg Bioanalyse^®^). After 24–48 h incubation at 27 °C, the diameter of the zones of inhibition were measured and interpreted according to the CLSI criteria [10].

### Data management and analysis

Questionnaire data was systematically checked for errors during data entry. All data obtained from the questionnaires were keyed into an Excel spread sheet (Microsoft Excel 2007 software) and cross checked for errors. It was then analyzed using the statistical package for social sciences (SPSS) version 20. To assess demographic, clinical and behavioral factors associated with onychomycosis, the logistic regression analysis was used to identify associated factors such as age, sex, duration and type of diabetes, presence of co-morbid diseases and family history of onychomycosis, occupation, footwear, habits of nail cutting. Odds ratios were used to report the association between onychomycosis and these associated factors. All statistical tests used were two-tailed, and values of P < 0.05 were considered statistically significant.

The strobe guideline for reporting observational studies was used in writing this manuscript [11].

## Results

Two hundred and twelve diabetics were approached, 152 met the inclusion criteria and where therefore enrolled. The participant ages ranged from 21 to 83 years (56.6 years ±SD = 12.1). The greater part of participants (54%) were aged 50–64 years. A vast majority were females (77%), 98% of study respondents were Type II diabetics and 49% had only a primary level of education.

The majority of the participants presented with mild nail damage 53.3% (n = 81), followed by moderate 27.6% (n = 42) and severe 19.1% (n = 29). Distal subungual onychomycosis was the most common clinical presentation recorded in 66.2%, followed by white superficial onychomycosis (16.9%), total dystrophic onychomycosis (10.4%) and endonyx onychomycosis (6.5%).

Out of the 152 diabetics who participated in the study 77 were confirmed for onychomycosis giving a prevalence of 50.7% (95% CI 42.4–58.9). Of the 77 who tested positive, 25% (n = 19) were by microscopy only, 34% (n = 26) by culture only and 41% (n = 32) for both microscopy and culture. The prevalence amongst diabetics with dystrophic nails was 53% (n = 59) and 45% (n = 18) in those with no nail dystrophy. The prevalence of onychomycosis with respect to severity of nail damage was as follows: mild (46.4%), moderate (64.7%) and (50%) for severe.

The association between demographic characteristics and onychomycosis is shown in Table 1. No factor assessed was significantly associated with onychomycosis in multiple logistic regression analysis. Notwithstanding, diabetics of age >64 years were about two times as likely to develop onychomycosis and those who had attained secondary education were about two times as likely to develop onychomycosis than those who had attained only primary education(OR = 0.61, 95% CI 0.21–1.82, P = 0.37).

**Table 1:** Demographic characteristics associated with onychomycosis in Diabetics in Fako

Characteristic	N° tested	% Positive	Odds ratio (95% CI)	P value
Age (years)				
19–34	9	33.3	1.00	–
35–49	23	47.8	1.83 (0.37–9.17)	0.46
50–64	82	51.2	2.10 (0.49–8.97)	0.31
>64	38	55.3	2.47 (0.54–11.37)	0.24
Sex				
Female	117	50.4	1.00	–
Male	35	51.4	1.04 (0.49–2.22)	0.91
Educational level				
Secondary	42	40.5	1.00	–
Primary	75	56.0	0.61 (0.21–1.82)	0.37
Higher	16	50.0	0.90 (0.24–3.41)	0.87
None	19	52.6	1.15 (0.42–3.14)	0.79

Table 2 shows the association between clinical characteristics and onychomycosis. None was statistically significant after multiple logistic regression analysis. Nonetheless diabetics with amputations and structural deformities were about two times as likely to develop onychomycosis (OR = 2.00, 95% CI 0.36–11.26, P = 0.43) when compared to those without.

**Table 2:** Clinical characteristics associated with clinical onychomycosis in diabetics in Fako

Characteristic	N° tested	% Positive	Odds ratio (95% CI)	*P* value
Duration of diabetes (years)				
<5	78	52.6	1.00	**–**
5–10	39	46.2	0.77 (0.36–1.67)	0.51
>10	35	51.4	0.96 (0.43–2.12)	0.91
Presence of foot ulcer				
No	150	50.7	1.00	–
Yes	2	50.0	0.97 (0.59–5.86)	0.98
Other co-morbid disease				
No	148	50.7	1.00	–
Yes	4	50.0	0.97 (0.13–7.09)	0.979
Amputation/structural deformity				
No	146	50.0	1.00	–
Yes	6	66.7	2.00 (0.36–11.26)	0.43
Family history of onychomycosis				
No	105	50.5	1.00	–
Yes	47	51.1	1.02 (0.51–2.04)	0.94
Nail trauma				
No	100	52.0	1.00	–
Yes	52	48.1	0.85 (0.44–1.67)	0.646

*Trichophyton* species were isolated as the causative agents of onychomycosis in diabetics in Fako.

**Fig. 1:**
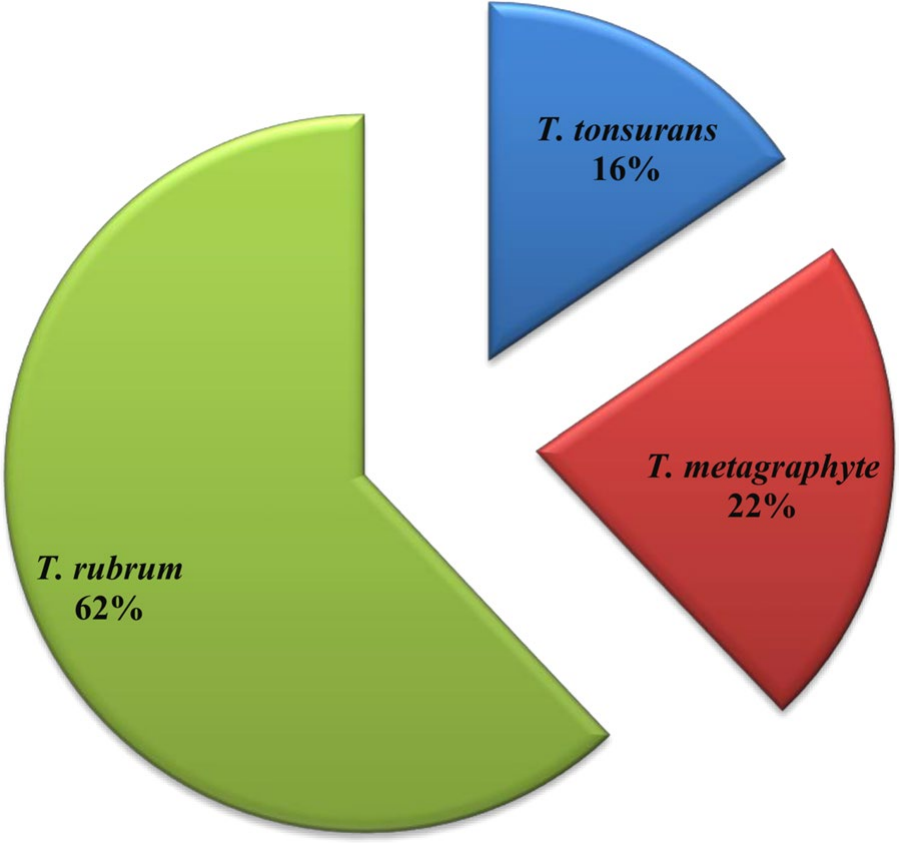
Dermatophyte distribution isolated from 58 diabetics with onychomycosis in Fako

*Trichophyton rubrum* was the most common causative agent isolated 36 (62%) followed by *T. metagraphyte* 13 (22%) and lastly *T. tonsurans* 9 (16%) (Fig. 1).

**Fig. 2:**
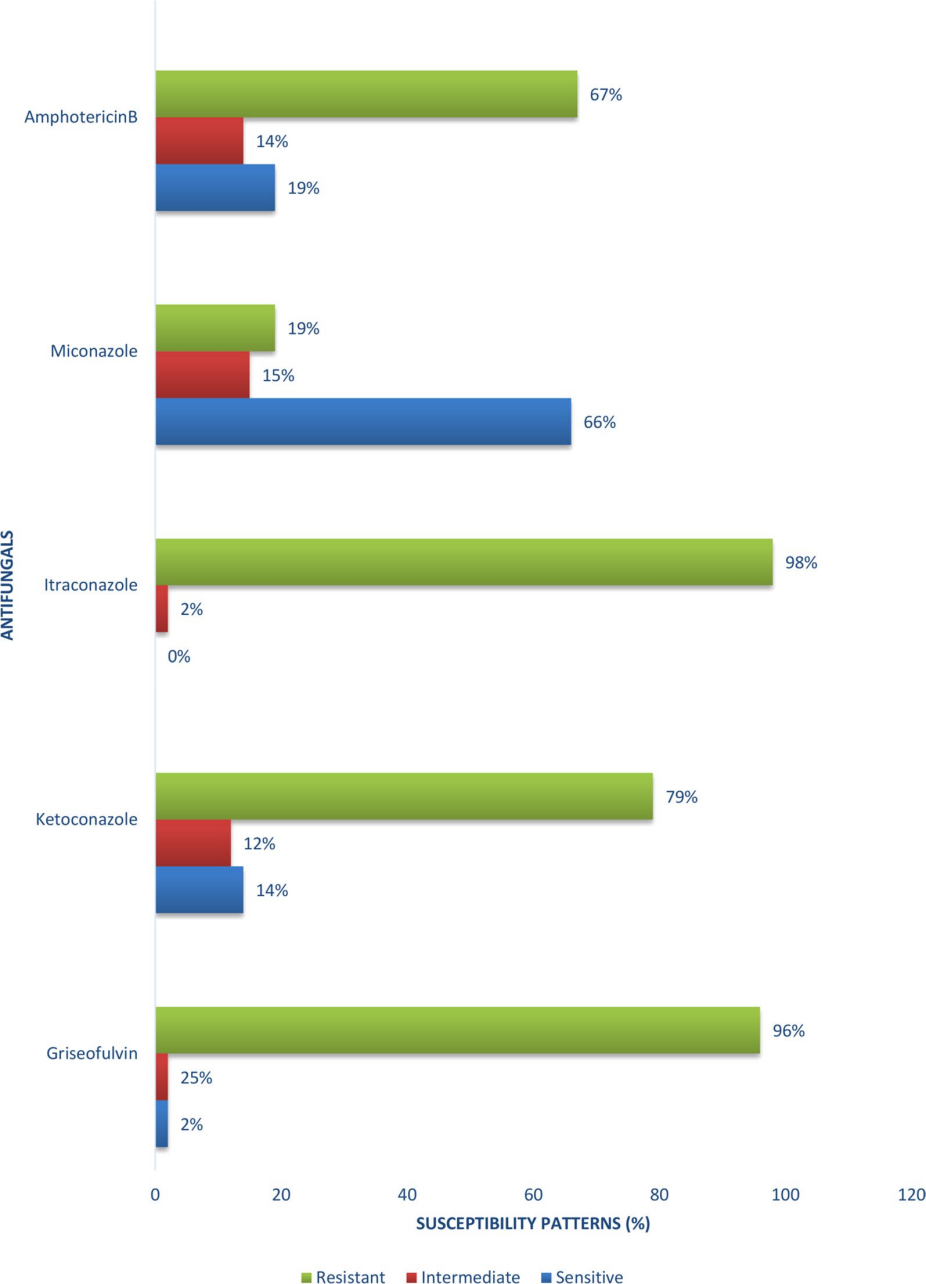
Antibiotic susceptibility patterns of *Trichophyton* isolates from 58 diabetics in Fako division, Cameroon

On the whole isolates were most sensitive to the azole miconazole (66%), the macrolide polyene amphotericin B (19%) and then the azole ketoconazole (14%). The least sensitive were griseofulvin (2%) and itraconazole (0%) (Fig. 2).

## Discussion

The prevalence of onychomycosis in diabetics in Fako was 50.7%. This prevalence is much higher than the 30% reported by Bouquerra et al. [12], in Tunisia and other researchers in countries where similar studies have been conducted [6, 13, 14]. This prevalence is nevertheless similar to the 50.4% obtained by Gerami et al. [15], and the 55% obtained by Sürücüoğlu et al. [16]. Other studies however indicated a higher prevalence than ours: 81% in Malaysia [17]. The relatively high occurrence of onychomycosis recorded could be accounted for by factors such as background illnesses, low socioeconomic status, poor hygienic conditions, crowded living conditions that provide multiple opportunities for disease transmission, immunodeficiency, quality of education given to patients at treatment centers and little or no examination of diabetics by a dermatologist. To the best of our knowledge, this is the first study to be performed among diabetics in Cameroon; however studies performed in the general population have revealed lower prevalence (8.8%) of onychomycosis [18]. Studies have shown that diabetic patients are at a higher risk of contracting onychomycosis compared to non-diabetics [4, 14]. In this study, distal subungual onychomycosis was observed to be the most common clinical presentation which is in line with studies performed elsewhere [14].

Factors associated with onychomycosis in diabetics identified in other studies [19–22] included sex, age, type and duration of diabetes, glycosylated haemoglobin levels for past three months, mean glucose levels for past 6 months, co-morbid diseases, family history, immunosuppressive therapy, structural amputation and deformities as well as socioeconomic status. In this study, no statistically significant associated factor of onchomycosis in diabetics was identified. This is in agreement with findings made by Süheyla et al. in Turkey [23]. Nonetheless diabetics of age greater than 64 years were about two times more likely to develop onychomycosis probably due to repeated nail micro-trauma, a more prolonged exposure to pathogenic fungi, decreased immunity, greater work activity and venous insufficiency. It was also noted that diabetics with amputations and structural deformities were about two times more likely to develop onychomycosis. The observation of higher risk of onychomycosis in elderly diabetic patients is in line with the study by Sigurgeirsson and Steingrímsson [24]. The observation of higher risk of contracting onychomycosis in patients who had attained secondary education to those who had attained only primary education could be due to the fact that the majority of participants in this study were in the strata of secondary education.

In this study we isolated dermatophytes as the causative agents of onychomycosis with, *T. rubrum* being most prevalent followed by *T. mentagrophytes* and lastly *T. tonsurans.* These findings are in concordance with previous studies conducted in diabetics [13, 25]. Other studies have shown non-dermatophyte molds [26] and yeasts [27] as the most common causative agents of onychomycosis. Studies performed on nondiabetics in other areas in Cameroon have reported non-dermatophytic molds and yeast as causative agent of onychomycosis, albeit lower frequency compared to dermatophytic molds [18, 28]. This discrepancy in distribution of etiologic agents could be due to geographical location and a wide range of environmental and cultural factors such as the use of common bathrooms in many homes and settlements, weighty manual work and agricultural practises that bring patients in contact with the soil, organic waste and dirty water. Dermatophytes thrive at surface temperatures of 25–28 °C and infection is supported by warm and high humid conditions [29]. This possibly explains the higher rates of infection with dermatophytes in Fako. Studies involving a larger number of diabetics will therefore be required in the study area to confirm this.

The azoles miconazole and ketoconazole were the most sensitive antifungals and this is in conformity with other studies [30, 31] that have proven desirable cure rates and even prevention of reoccurrence obtainable through the use of these drugs. In addition isolates of *T. rubrum* and *T. metagraphyte* were also sensitive to amphotericin B. This conflicts report given by Zaias et al. and Gupta et al. who obtained high clinical and mycological cure rates in patients treated with itraconazole and griseofulvin [29, 31]. The high resistance to itraconazole obtained is similar to that reported by Hryncewicz et al. [32], who observed resistance in 80% of isolates tested in Poland. High resistant rates to griseofulvin also conforms to that stated by Elewski who recorded high rates of resistance and patient relapse when treated with griseofulvin [33].

In this study we did not identify any factor associated with onychomycosis probably due to the relatively small sample size. Secondly other factors which could possibly influence onychomycosis as glycosylated haemoglobin levels for the past three months and mean glucose levels for the past 6 months were not assessed. The results obtained from the study cannot be generalized to the entire Cameroon as the study was limited only to diabetics in Fako division.

We suggest that further research be carried out in other parts of the country integrating the factors left out so as to establish factors common in our region and other regions of the country.

## Conclusions

Our study suggests that approximately one out of every two diabetic in Fako may have onychomycosis and this could be detrimental to their health as it could lead to diabetic foot and amputations. We did not identify any statistically significant associated factor of onychomycosis amongst this patient population. *T. rubrum* was the most common dermatophyte isolated and isolates were most sensitive to miconazole, amphotericin B and ketoconazole. All isolates of *T. tonsurans and T. metagraphyte* were completely resistant to itraconazole and griseofulvin.

## Additional file

**Additional file 1: Questionnaire.** The questionnaire used to collect demographic, behavioural and clinical data.

## Abbreviations

RHB: Regional Hospital of Buea
RHL: Regional Hospital of Limbe
SDA: Sabouraud Dextrose Agar
KOH: Potassium hydroxide
