# Supplementary material for: Onychomycosis in diabetic patients in Fako Division of Cameroon: prevalence, causative agents, associated factors and antifungal sensitivity patterns
Source: BMC Res Notes. 2016 Nov 22;9:494. doi: 10.1186/s13104-016-2302-1 (PMC5120551; doi:10.1186/s13104-016-2302-1)
Supplement: Supplementary file 1 — Additional file 1: Questionnaire. The questionnaire used to collect demographic, behavioural and clinical data. [file 13104_2016_2302_MOESM1_ESM.docx]

**QUESTIONNAIRE**

Thank you for accepting to take part in this study on onychomycosis. As a participant, you are required to complete this questionnaire containing short answer questions, which will take about 5 minutes to complete. When you are through, return the questionnaire to the dermatologists will examine you. Reserve section B of this questionnaire for use by the dermatologist.

NB: keep the responses strictly confidential. Enclose the questionnaire in the envelope provided.

**SECTION A: FOR USE BY THE PARTICIPANT**

**Name:** ____________________________________________________________________

**Age:** ______________

**Gender:** M F

**Where do you live?** __________________________

1. Have you been on any drug within the past 2 weeks? Yes No
2. Are you currently on any drug? Yes No
3. Was the drug prescribed by a doctor? Yes No
4. The drug was prescribed to treat which disease? _________________________________
5. What is the name of the drug? _______________________________________________
6. How long have you been suffering from diabetes? ____________
7. Are you pregnant? Yes No

**If No, skip to question 10.**

1. Were you send to the diabetic clinic because your sugar level was high during the pregnancy? Yes No
2. What was the value of your blood sugar? _____________
3. Do you have nail infection on your fingers or toes? Yes No
4. Are rubbing any cream or taking any drug to treat the infection? Yes No
5. Do you have any member of your family with nail infection? Yes No
6. Are you HIV positive? Yes No
7. List any other disease you are suffering from ____________________________________

I certify that all the information provided in here are true.

Signature of the participant _______________________ Date_________________________

**SECTION B: TO BE COMPLETE BY THE DERMATOLOGIST**

Type of diabetes the patient is suffering from: _____________________________

Presence of nail lesion or trauma: Yes No

Presence of foot ulcer: Yes No

Presence of amputation or structural deformity: Yes No

For participants with visible nail damage, choose a classification

Distal subungual onychomycosis

Total dystrophic onychomycosis

White superficial onychomycosis

Endonyx onychomycosis

Candida paronychia

Signature of dermatologist _________________________ Date ____________________
